# Hybrid Microscopy: Enabling Inexpensive High-Performance Imaging through Combined Physical and Optical Magnifications

**DOI:** 10.1038/srep22691

**Published:** 2016-03-15

**Authors:** Yu Shrike Zhang, Jae-Byum Chang, Mario Moisés Alvarez, Grissel Trujillo-de Santiago, Julio Aleman, Byambaa Batzaya, Vaishali Krishnadoss, Aishwarya Aravamudhan Ramanujam, Mehdi Kazemzadeh-Narbat, Fei Chen, Paul W. Tillberg, Mehmet Remzi Dokmeci, Edward S. Boyden, Ali Khademhosseini

**Affiliations:** 1 Biomaterials Innovation Research Center, Division of Biomedical Engineering, Department of Medicine, Brigham and Women’s Hospital, Harvard Medical School, Boston 02139, MA, USA; 2 Harvard-MIT Division of Health Sciences and Technology, Cambridge 02139, MA, USA; 3 Wyss Institute for Biologically Inspired Engineering, Harvard University, Boston 02115, MA, USA; 4 Media Lab, MIT, Cambridge 02139, MA, USA; 5 Centro de Biotecnología-FEMSA, Tecnológico de Monterrey at Monterrey, CP 64849, Monterrey, Nuevo León, México; 6 Microsystems Technologies Laboratories, MIT, Cambridge, 02139, MA, USA; 7 School of Chemical & Biotechnology, SASTRA University, Tamil Nadu 613401, India; 8 Department of Biological Engineering, MIT, Cambridge 02139, MA, USA; 9 Department of Electrical Engineering and Computer Science, MIT, Cambridge 02139, MA, USA; 10 McGovern Institute, MIT, Cambridge 02139, MA, USA; 11 Department of Brain and Cognitive Sciences, MIT, Cambridge 02139, MA, USA; 12 Center for Neurobiological Engineering, MIT, Cambridge 02139, MA, USA; 13 Department of Bioindustrial Technologies, College of Animal Bioscience and Technology, Konkuk University, Hwayang-dong, Gwangjin-gu, Seoul 143-701, Republic of Korea; 14 Department of Physics, King Abdulaziz University, Jeddah 21569, Saudi Arabia

## Abstract

To date, much effort has been expended on making high-performance microscopes through better instrumentation. Recently, it was discovered that physical magnification of specimens was possible, through a technique called expansion microscopy (ExM), raising the question of whether physical magnification, coupled to inexpensive optics, could together match the performance of high-end optical equipment, at a tiny fraction of the price. Here we show that such “hybrid microscopy” methods—combining physical and optical magnifications—can indeed achieve high performance at low cost. By physically magnifying objects, then imaging them on cheap miniature fluorescence microscopes (“mini-microscopes”), it is possible to image at a resolution comparable to that previously attainable only with benchtop microscopes that present costs orders of magnitude higher. We believe that this unprecedented hybrid technology that combines expansion microscopy, based on physical magnification, and mini-microscopy, relying on conventional optics—a process we refer to as Expansion Mini-Microscopy (ExMM)—is a highly promising alternative method for performing cost-effective, high-resolution imaging of biological samples. With further advancement of the technology, we believe that ExMM will find widespread applications for high-resolution imaging particularly in research and healthcare scenarios in undeveloped countries or remote places.

Optical microscopy became an indispensable technique in biomedicine following its invention in the 16^th^ century. Tremendous effort has since been invested on enhancing optical microscopy instrumentation. For example, several modalities, such as phase contrast (PHC) microscopy and differential interference contrast (DIC) microscopy, have used interference to improve contrast when viewing cellular structures^1^,^2^. The development of confocal and multi-photon microscopy has enabled volumetric imaging of biological samples in three dimensions with minimum invasiveness^3^,^4^. These improvements have furthered the demand for techniques that allow viewing subcellular interactions at the organelle or molecular scales, thereby fostering the development of super-resolution microscopy based on complex optics and/or algorithms^5^,^6^,^7^,^8^,^9^,^10^,^11^,^12^,^13^,^14^,^15^.

Contrarily, instrumentation has also been pushed to the other end by development of miniature imagers featuring low complexity and cost^16^,^17^,^18^,^19^,^20^. These mini-microscopes are usually targeted for use in high-throughput and *in situ* analysis of multiple units in parallel and are appropriate for research and healthcare scenarios where portability and cost-effectiveness are prerequisites. Several compact image sensors based on lens-free technology are now available; for example, a lens-free imaging system has been used for real-time beating analysis of neonatal cardiomyocytes upon treatment with cardiotoxic drugs^18^. Mini-microscopes have also been applied to point-of-care pathology and diagnosis^21^,^22^,^23^,^24^. We have recently developed a miniature fluorescence microscope with adjustable magnifications for universal mounting and imaging of biological structures at a cost as low as a few dollars per piece^25^. However, the resolution of the resulting images (including our mini-microscope and low-cost portable imagers in general) is usually limited, which is insufficient for high-resolution analysis, and raising the question of whether it is possible to achieve extremely high performance microscopy with very low cost.

Expansion microscopy (ExM) is a recently developed technique^26^ that enables nanoscale resolution imaging without specific high-end hardware. Unlike other approaches, ExM relies on a very simple principle; namely, embedding a biological sample in a homogeneous matrix of swellable gel, which chemically anchors fluorescent labels applied to the specimen, followed by mechanical homogenization of the sample and physical expansion of the sample by swelling the gel-specimen composite to up to approximately 4.5 times its original dimensions^26^. In this way, ExM effectively improves the diffraction-limited resolution by a factor of 4.5, even achieving nanoscale resolution imaging without the need to modify the existing microscope hardware.

Here we have further develop a “hybrid microscopy” method—combining physical and optical magnifications—that we here refer to as ‘Expansion Mini-Microscopy’, or ExMM. We have demonstrated that the physical expansion of a biological sample and imaging the sample using our mini-microscope achieves a resolution comparable to, if not even higher than, that attainable by the conventional benchtop microscope prior to expansion, at hundred-to-thousand-fold reduction in price. We anticipate that our ExMM technique is likely to find widespread applications in low-cost, high-resolution imaging of biological and medical samples, potentially replacing the conventional benchtop microscope in many cases where portability is a priority. This ExMM technology may also be useful in point-of-care diagnosis in developing countries or resource-limited regions by expanded-scale imaging of pathogens that are otherwise barely visible using the low-cost mini-microscopes.

## Results and Discussion

**Figure 1:**
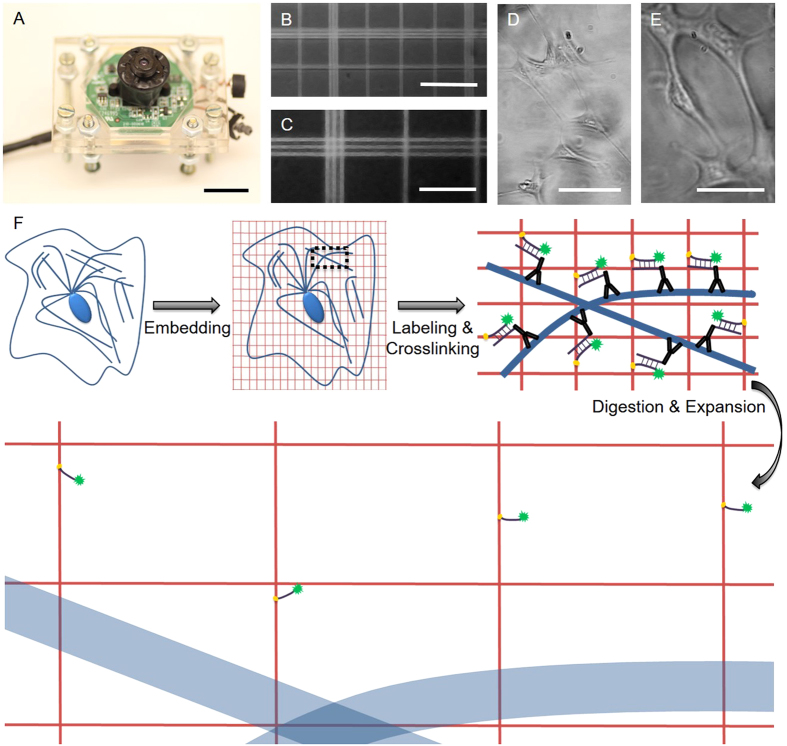
Concepts of mini-microscopy and expansion microscopy. (**A**) Photograph showing the mini-microscope; scale bar: 5 mm. (**B**,**C**) Mini-microscope images of a hemocytometer at 8X and 20X, respectively; scale bars: 100 μm and 50 μm. (**D**,**E**) Mini-microscope images of NIH/3T3 fibroblasts at 8X and 20X, respectively; scale bars: 200 μm and 100 μm. (**F**) Principle of expansion microscopy (ExM).

The construction of the mini-microscope was based on our recently published protocol using a webcam and off-the-shelf components, such as poly(methyl methacrylate) (PMMA) boards, screw/bolt sets, and broadband/monochromatic LEDs^25^. The mini-microscope body was composed of a CMOS sensor and an inverted webcam lens for magnifying the samples (Fig. 1A). By controlling the distance between the lens and the CMOS sensor, the magnification of the mini-microscope could be tuned anywhere between 8X (6.5 mm) to 60X (48 mm). Mini-microscopy images of a hemocytometer at 10X and 20X, and of a resolution target, revealed a resolution of approximately 1–2 μm (Fig. 1B,C and Supplementary Fig. 1). NIH/3T3 fibroblasts were also clearly imaged at both magnifications with individual cells discernible (Fig. 1D,E). The axial resolution of the mini-microscope was characterized to be approximately 34 μm at 20X, as measured by the full width half maximum (FWHM) from the point spread function (Supplementary Fig. 2). The aberration of the images obtained from our mini-microscope was analyzed, which showed that the distortions originated from the mini-microscope were small in scale and negligible in all directions (<1%). High-sensitivity fluorescence imaging in the green fluorescent protein (GFP) channel was enabled with the mini-microscope by employing a monochromatic LED (wavelength: 490 nm) as the excitation source and adopting a long-pass filter of >530 nm to separate the emission energy from the excitation or digital channel separation^25^.

The principle of ExM is based on physical expansion of the specimen to achieve magnification and improved resolution (Fig. 1F). Subcellular features (e.g. microtubules) are first labeled with a customized fluorescent probe that carries a methacryloyl group capable of participating in free radical polymerization. Subsequent infiltration of a pre-polymer solution containing a monomer, sodium acrylate (as well as an acrylamide co-monomer), an N-N’-methylenebisacrylamide crosslinker, an ammonium persulfate (APS) initiator, and a tetramethylethylenediamine (TEMED) accelerator, trigger the chemical gelation of the sample and gel-anchoring of the fluorescent labels. Treatment of the construct with a proteolytic enzyme, to achieve controlled degradation of the biological structure without destroying the anchored fluorophore, then result in homogenous mechanical properties of the specimen. The last step—immersion in water—results in a 4.5-fold linear osmotic expansion of the sample with ~1% geometrical distortion at macro- and micro-scales (down to the resolution of tens of nanometers). Importantly, the digested and expanded specimen is also optically transparent due to dilution of the sample, as it becomes >99% water during the expansion process, and structures that scattered light are now absent. This sample processing protocol is compatible with the conventional and widely adopted immunostaining techniques, which also mainly use fixed/permeabilized samples for labeling specific biomarkers of interest. In addition, the cost of the reagents required to use this technique is minimal at a low-volume setting. For instance, at a working volume of 100 μL, which is sufficient for observation using the mini-microscope, the estimated cost for all the reagents would be less than $2.50 USD (Supplementary Table 1).

**Figure 2:**
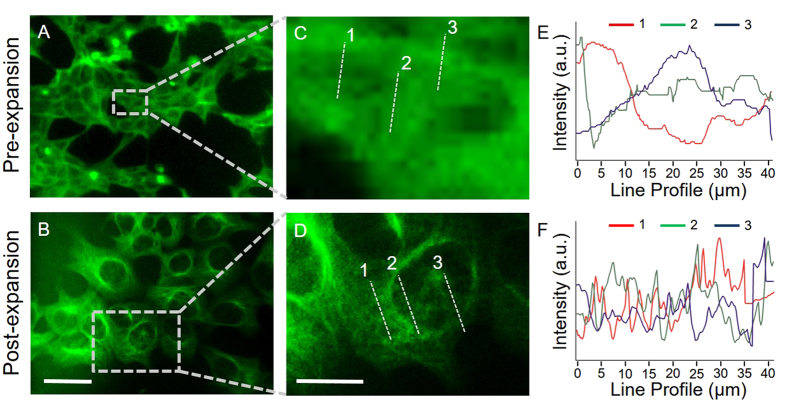
ExMM imaging of tubulin for HEK cells. (**A**,**B**) Pre- and post- expansion images obtained with a mini-microscope; scale bar: 100 μm. (**C**,**D**) Magnified views of the selected regions in (**A**,**B**); scale bar: 40 μm. (**E**,**F**) Intensity profiles of dotted lines taken at three different locations in (**C**,**D**), respectively.

We first examined the capability of expansion to improve the resolution of the mini-microscope. We stained a monolayer culture of human embryonic kidney (HEK) 293 cells for tubulin in green fluorescence, and then compared the mini-microscope images pre- and post-expansion (Fig. 2). A pre-expansion mini-microscope image showed clearly distinguished individual cells with vaguely discernable nuclei (Fig. 2A). In contrast, physical expansion of the sample to 4.5 times the original dimensions caused the nuclei to become clearly distinguishable from the cytoplasm (Fig. 2B). The magnified images (Fig. 2C,D) showed no tubulin structures in the pre-expansion image (Fig. 2C), whereas traces of tubulin fibrils were visible in the mini-microscope image taken after the expansion of the sample (Fig. 2D). We also quantified the intensity profiles along the lines indicated in respective images, and plotted these against distance (Fig. 2E,F). The subcellular features were much better resolved in the post expansion mini-microscope images than in the pre-expansion images, as indicated by the more densely spaced peaks along the distance (Fig. 2F). Without expansion, however, only very rough features could be discerned (Fig. 2E). Clearly, the expansion technique works well independent of the microscope used for imaging and lends itself successfully to mini-microscopy, in a process that we refer to as ExMM. The technique described here confirmed an improved resolution and applicability to a low-cost mini-microscope without the need for additional instrumentation or significant modification of ExM. According to the resolution measured for our mini-microscope (1–2 μm), a 4.5-fold expansion would increase the resolution of ExMM to approximately 250–500 nm, which is close to that achievable with high-quality optical microscopes.

**Figure 3:**
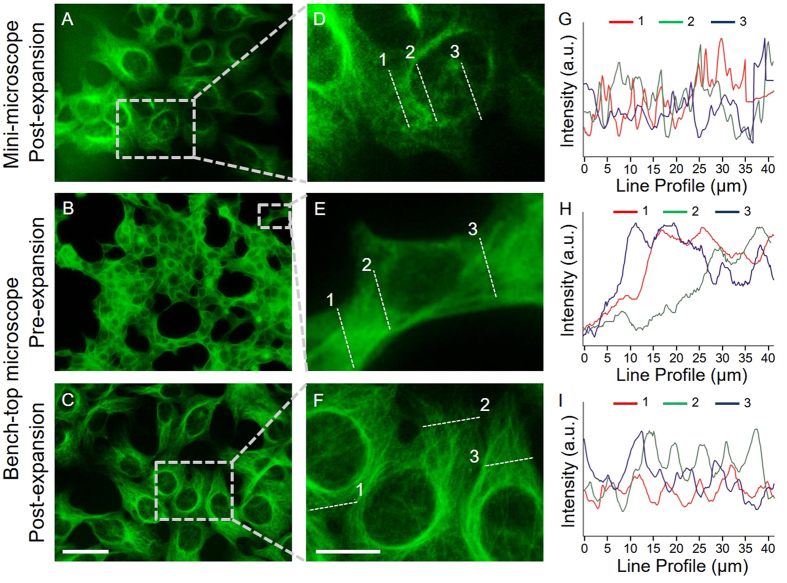
Comparison between ExMM and ExM. (**A**) Post-expansion image obtained with a mini-microscope. (**B**,**C**) Pre- and post-expansion images obtained with a benchtop microscope. Objective: 10X, NA = 0.25. (**D–F**) Magnified views of the selected regions in (**A–C**). (**G–I**) Intensity profiles of dotted lines taken at three different locations in (**D–F**), respectively. Scale bars: 100 μm for (**A–C**); 40 μm for (**D–F**).

We examined the efficacy of the ExMM further by comparing it with that of ExM (Fig. 3). In the post-expansion mini-microscope images of HEK 293 cells stained for tubulin (Fig. 3A,D), the nuclei were clearly observable and the microtubule structures were also visible. Interestingly, without expansion, even the much more sophisticated benchtop microscope could barely distinguish the microtubules in the cells, although the shapes of the nuclei showed up better than they did in the pre-expansion images obtained with the mini-microscope (Fig. 3B,D *versus* Fig. 2B,D). As expected, the benchtop microscope image of the cells post expansion had significantly improved resolution and the individual microtubules were visible. Intensity profiles of the lines indicated in the images were further quantified (Fig. 3G–I). The images obtained from the mini-microscope seemed to be more speckled when compared to those from the benchtop microscope, which was most likely due to the much lower sensitivity of the low-cost mini-microscope compared with the multi-thousand benchtop microscope. The magnification achieved by the EXMM via physical expansion of the specimen clearly provided a resolution nearly comparable to that of the costly benchtop microscope for specimens undergoing the same expansion, but, intriguingly, the resolution of the ExMM seemed even higher than the pre-expansion resolution of the benchtop microscope in this case, as observed from the images. This finding is very attractive; we have shown that the combination of ExM with a mini-microscope provided by our ExMM technology could result in a resolution that rivals that of a conventional microscope with a hundred-to-thousand-fold reduction in the cost.

**Figure 4:**
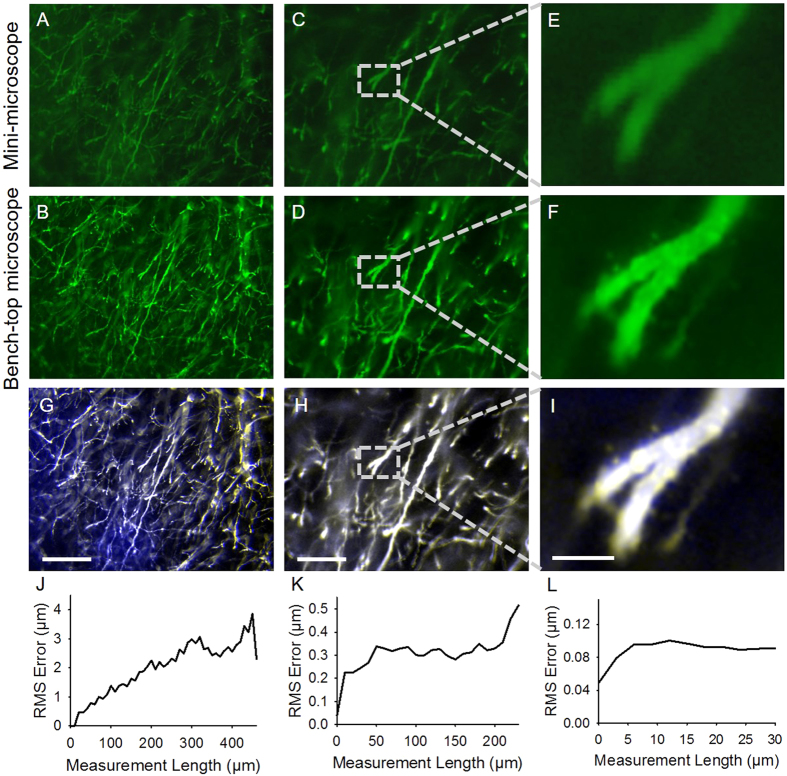
Comparison between ExMM and ExM of expanded mouse brain slice. (**A–D**) Images obtained with a mini-microscope (**A**,**C**) and with a bench-top microscope (**B**,**D**) at 10X (NA = 0.25) (**A**,**B**) and 20X (NA = 0.4) (**C**,**D**) magnifications. (**E**,**F**) Magnified views of the selected regions in (**C**,**D**), respectively. (**G–I**) Rigid co-registration of ExMM and ExM images in (**A**–**F**). Blue: ExMM images; white: ExM images. (**J–L**) Calculated RMS errors of ExMM and ExM images using a non-rigid co-registration process. Scale bars: 200 μm for (**A**,**B**,**G**); 100 μm for (**C**,**D**,**H**); 20 μm for (**E**,**F**,**I**).

The correlation between ExMM and ExM was subsequently investigated by imaging expanded mouse brain slices in the same location using the mini-microscope and a benchtop microscope (Fig. 4). Brain slices from genetically engineered mice expressing yellow fluorescent protein (YFP) in a subset of neurons were stained with antibodies to YFP in green fluorescence, and imaged using ExMM (Fig. 4A,C) and ExM (Fig. 4B,D) at the same location of a sample at 10X (Fig. 4A,B) and 20X (Fig. 4C,D) magnifications. The two sets of images obtained by ExMM and ExM were then subjected to rigid co-registration (Fig. 4G–I). The majority of the features in each pair of post-expansion images obtained with the mini-microscope and the benchtop microscope showed co-localization, indicating the good resolution of the ExMM when compared with ExM. Images obtained at both 10X and 20X magnifications (Fig. 4G,H) revealed good matching of the center of the images, while most mismatches happened at the periphery, which however, were still very small. Indeed, the magnified view at the center of the 20X-image, after rigid co-registration, showed minimal aberration and led to almost completely matching features (Fig. 4I).

We quantified the distortion of ExMM by calculating the deformation vector fields between the ExMM and ExM images via a non-rigid registration process, as done before to validate the original ExM protocol^26^. These vector fields were then used to quantify the root-mean-square (RMS) error of feature measurements in registered images (see Supplementary Fig. 3 for details)^26^. The calculated RMS errors were relatively small (<1% of measurement lengths) in all cases (Fig. 4J–L), again confirming the remarkable ability of the ExMM as a technique that enables high-resolution, high-accuracy imaging of biological samples at low cost. This observation was consistent with our initial aberration analysis, which showed that the distortions of the images obtained from the mini-microscope were negligible in all directions (<1%).

**Figure 5:**
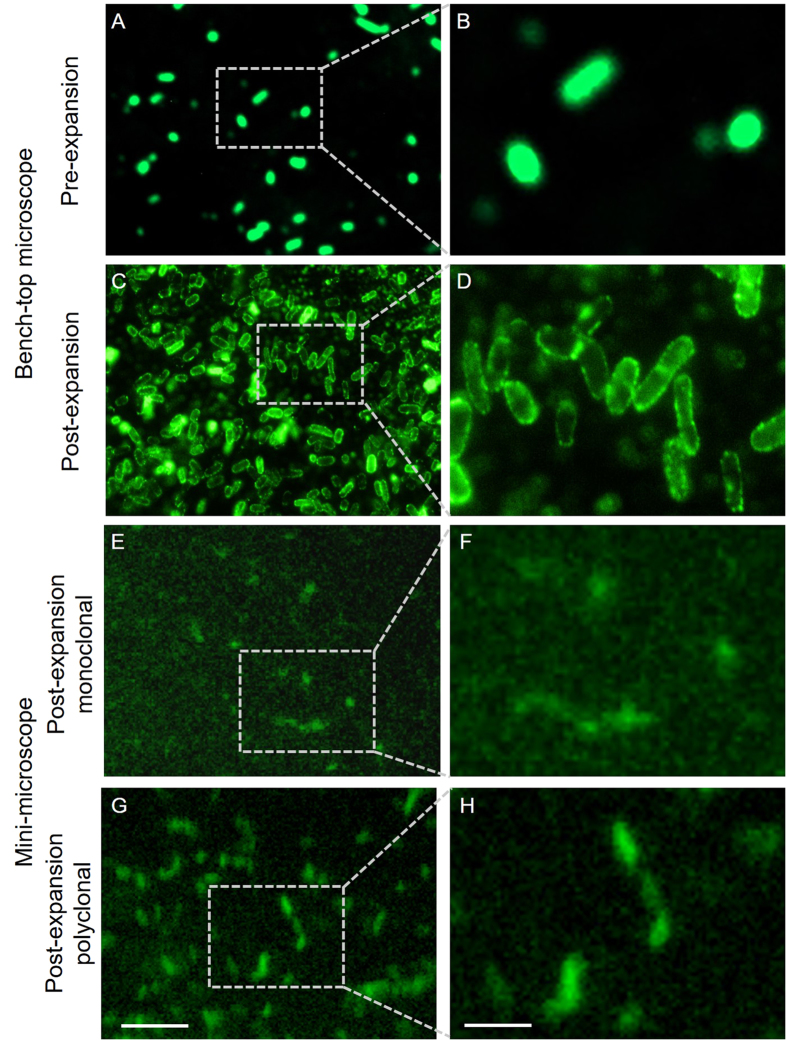
Comparison between ExMM and ExM of *E. coli*. (**A**,**B**) Pre-expansion images obtained with a benchtop microscope. (**C**,**D**) Post-expansion images obtained with a benchtop microscope. Objective: 20X, NA = 0.4. (**E**,**F**) Post-expansion images obtained with a mini-microscope, where bacteria were stained using a monoclonal antibody to lipopolysaccharide. (**G**,**H**) Post-expansion images obtained with a mini-microscope, where bacteria were stained using a polyclonal antibody to *E. coli*. Scale bars: 5 μm for (**A**,**C**,**E**,**G**); 2 μm for (**B**,**D**,**F**,**H**).

We further demonstrated the utility of ExMM by extending its application to point-of-care technologies in the detection of pathogens such as bacteria, which are typically challenging to observe under miniature microscopy due to their small sizes. We labeled *Escherichia coli* (*E. coli*) with an antibody that targets lipopolysaccharides on the membrane of the bacteria (Fig. 5A,B). The bacteria were then embedded in the hydrogel and expanded approximately 4.5 times following a similar procedure as used for expansion of the HEK cells. After expansion, the bacterial cells showed much clearer morphology with the benchtop microscope (Fig. 5C,D). Importantly, the presence of *E. coli* (stained by the monoclonal anti-lipopolysaccharide primary antibody) could only be imaged using the mini-microscope in post-expansion samples (Fig. 5E,F), whereas no deterministic signals were detected from the bacteria in their original unexpanded state due to the tiny size of these bacteria with individual cells measuring about only 0.5 μm (width) ×2 μm (length) and the relatively low sensitivity of the low-cost mini-microscope. It was further found that, the signal-to-noise ratio of the bacteria images could be significantly improved by switching to a polyclonal antibody prepared against mixture of different *E. coli* serotypes (Fig. 5G,H). While it required long exposure (>8–10 s depending on the field-of-view) of the mini-microscope to image the bacteria stained with monoclonal antibodies, such exposure time was reduced to (2–4 s) for *E. coli* stained with polyclonal antibodies. In addition, the shapes of the bacteria were more clearly discernible in the polyclonal version of the images (Fig. 5G,H) than the monoclonal version (Fig. 5E,F), indicating at least a 4–5 times of improvement in the brightness of the fluorescence. By targeting different membrane epitopes, a polyclonal cocktail of antibodies enables a more homogeneous coverage of the bacterial membrane and therefore provides a greater resolution of the membrane. In both cases, the contours of the *E. coli* in the ExMM images were not as clearly defined as those seen in the benchtop microscope images. This observation can be reasonably explained by the lower number of pixels (1.2 million) in the mini-microscope compared to that for the benchtop microscope, together with the limited sensitivity of the mini-microscope. The poor axial resolution (34 μm, Supplementary Fig. 2) of the mini-microscope might have also contributed to the decreased clarity of the contours particularly for samples of small sizes. However, the capability of our ExMM technology to identify these small-sized bacteria should suffice for most point-of-care applications. We believe that the ExMM provides a unique and highly cost-effective approach for the diagnosis of bacteria-induced diseases, which could be particularly useful in remote regions of the world.

It should be noted, however, that the reduced density of fluorophores in the post-expansion specimen require a much longer exposure time, due to the relatively low-sensitivity CMOS sensor of the mini-microscope, than is needed for the benchtop microscope. In general, the exposure time required for the ExMM typically exceeds a few seconds, thereby resulting in strong background noise (Supplementary Fig. 4A). By comparison, the benchtop microscope obtains much cleaner images with minimal need for image processing (Supplementary Fig. 4C). However, this minor drawback of the ExMM could be easily amended by linear contrast enhancement of the images to remove the background signals and retrieving the structural information of the specimens (Supplementary Fig. 4B). In fact, the sensitivity achievable by ExM/ExMM is partially a function of the concentration of the antibodies used to tag the subcellular structures in a specimen. Consequently, the use of a higher concentration of the primary antibody will likely enable higher sensitivity of ExMM imaging. Alternatively, the use of antibody fragments of smaller sizes (e.g. nanobodies^27^,^28^,^29^) should also conceptually improve the quality of the post-expansion image by tagging the target structure of a sample with a higher density of fluorophores, as would amplification methods to augment brightness post-expansion.

We note the current limitation of the ExMM technology regarding the dependence on the longer sample preparation protocols. Such protocols require approximately twice the time as conventional immunostaining methods due to the involvement of the gelation process necessary for subsequent expansion. However, considering the portability and the significantly reduced cost in the instrumentation (the mini-microscope of few to few tens of dollars *versus* the multi-thousand-dollar benchtop microscope), we regard our hybrid ExMM approach as presenting a valuable and cost-effective alternative. The further advancement of the technology, through the development of an ExMM detection kit that integrates a disposable device for automated sample labeling and expansion, the mini-microscope, and the algorithms associated with imaging processing, will allow streamlined sample preparation, imaging, and analysis. This type of kit, once developed, will require a minimum level of technical expertise when compared to that required for the implementation and interpretation of results using current diagnostic techniques. Moreover, the cost of the reagents will be significantly reduced with the development of a kit with miniaturized volumes (a few μL). Therefore, we believe that our ExMM will likely find widespread applications for low-cost, high-resolution imaging, particularly in research and healthcare scenarios in undeveloped countries or resource-limited regions.

## Methods

### Mini-microscope

The mini-microscope was constructed by modifying our recently published protocol^25^. A commercial Logitech webcam was disassembled to obtain the CMOS sensor. The base structure of the mini-microscope was produced from a PMMA sheet by laser cutting (VLS 2.30 Desktop Laser System, Universal Laser Systems), and assembled using screw/bolt pairs. The bottom PMMA frames held the CMOS and lens, while the top frame allowed the positioning of a glass slide for sample imaging. Four additional sets of screws/bolts were mounted at the edge between the sample holder and the base for convenient focal adjustment.

The system was converted into a mini-microscope by inverting the lens and re-attaching it to the CMOS sensor to achieve magnification. We obtained 10X and 20X magnifications by cutting cylinders with heights of 6 mm and 12 mm from 2.0-mL Eppendorf tubes, which functioned as spacers between the lens and the CMOS. These tubes were wrapped in a dark tape to prevent interference from the ambient light in the environment. Images were acquired with a computer using custom-coded MATLAB (Mathworks) programs by connecting the camera through the USB port^25^.

### DNA-labeled secondary antibody and tri-functional label preparation

Short oligonucleotides with 5′ amine modification were synthesized (Integrated DNA Technologies) and conjugated to donkey anti-sheep secondary antibody, donkey anti-mouse secondary antibody, and donkey anti-chicken secondary antibody (Jackson ImmunoResearch) using a commercially available conjugation kit (AntibodyOligonucleotide All-in-One Conjugation Kit, Solulink,). The sequence of the oligonucleotides was 5′amine-CCG AAT ACA AAG CAT CAA CGA AGG TGA CAG GGA TCA CAA TCT-3′. Two dual-functional oligonucleotides with 5′ acrydite and 3′ Alexa 488 modifications were synthesized (Integrated DNA Technologies) as complimentary sequences to the secondary antibody-conjugated oligonucleotides to construct the tri-functional labels. The sequences of the two dual-functional labels were 5′ acrydite-CGT TGA TGC TTT GTA TTC GG-3′ Alexa488 and 5′ acrydite-AGA TTG TGA TCC CTG TCA CC-3′ Alexa488.

### Cultured cell sample preparation and staining

HEK293-FT human embryo kidney cells (Life Technologies) were maintained in a Dulbecco’s Modified Eagle Medium (DMEM, Life Technologies) supplemented with 10 vol.% heat-inactivated fetal bovine serum, 1 vol.% penicillin and streptomycin, and 1 vol.% sodium pyruvate and incubated at 37 °C in a humidified atmosphere containing 5 vol.% CO_2_. The cells were plated on chambered cover glasses (Life Technologies) 1–2 days prior to fixation. The cells were then subjected to fixation in 3 vol.% formaldehyde and 0.1 vol.% glutaraldehyde in 1X phosphate buffered saline (PBS) for 10 min, and then reduced with 0.1 vol.% NaBH_4_ in 1X PBS for 7 min. The cells were rinsed three times with 0.1 M glycine in 1X PBS, for 10 min each time. The cells were then permeabilized with 0.2 vol.% Triton X-100 in 1X PBS for 15 min and blocked with 5 vol.% normal donkey serum (Life Technologies) in 1X PBS (i.e. the blocking buffer) for 15 min. The cells were stained with sheep anti-tubulin antibody (ATN02, Cytoskeleton) in the blocking buffer at a concentration of 10 μg mL^−1^ for 2 h at room temperature, followed by washing in 1X PBS three times, for 5 min each time. The cells were then stained with DNA-labeled donkey anti-sheep secondary antibody in 2X saline-sodium citrate buffer, 10% dextran sulfate, 1 mg mL^−1^ yeast tRNA, and 5 vol.% normal donkey serum (i.e. the hybridization buffer) at a concentration of 10 μg mL^−1^ for 4 h at room temperature and then washed in 1X PBS. The cells were then incubated overnight with the two dual-functional labels in the hybridization buffer at a concentration of 0.5 ng μL^−1^, and then washed three times with 1X PBS.

### Cultured *E. coli* sample preparation and staining

*E. coli* in the mid logarithmic phase of growth was obtained by transferring 100 mL of an overnight culture of the bacteria into sterile tubes containing 5 mL of Mueller-Hinton Broth (MHB, Sigma-Aldrich) and incubating at 37 °C for 1 h. The bacteria suspensions were immediately centrifuged and washed in PBS, followed by fixation in 70 vol.% ethanol in PBS at −20 °C for 15 min and triple washes with PBS. The bacteria were then permeabilized with 1 vol.% Triton X-100 in 1X PBS at room temperature for 15 min, blocked in 1 wt.% bovine serum albumin (BSA) 1X PBS for 15 min, and incubated overnight at 4 °C with the primary anti-lipopolysaccharide monoclonal antibody (ab35654, Abcam, 1:50) or the anti-*E. coli* polyclonal antibody (ab137967, Abcam, 1:50) in 1 wt.% BSA in 1X PBS. After the primary antibody staining, the bacteria were washed three times in 1X PBS and then stained with DNA-labeled donkey anti-mouse secondary antibody in the hybridization buffer at a concentration of 10 μg mL^−1^ for 4 h at room temperature and then washed in 1X PBS. The bacteria were then incubated overnight with the two dual-functional labels in the hybridization buffer at a concentration of 0.5 ng μL^−1^, and then washed three times with 1X PBS.

### Brain slice sample preparation and staining

All mice brain tissue experiments reported here were conducted using transgenic mice expressing cytosolic YFP under the Thy1 promoter (Thy1-YFP-H strain on C57BL/6, obtained from the Jackson Laboratory). All procedures involving animals were in accordance with the US National Institutes of Health Guide for the Care and Use of Laboratory Animals and approved by the Massachusetts Institute of Technology Committee on Animal Care. The mice were anesthetized with isoflurane and perfused transcardially with ice-cold 4 vol.% paraformaldehyde in 1X PBS. Brains were dissected out and incubated in 4 vol.% paraformaldehyde in 1X PBS at 4 °C for 1 day, and then immersed in 30 vol.% sucrose with 100 mM glycine in 1X PBS for another day. Brains were sliced to a 100 μm thickness using a vibratome (Leica VT1000S). The 100-μm-slices were incubated at room temperature for 12 h with chicken anti-YFP antibody (AB16901, Abcam) diluted to 10 μg mL^−1^ in 0.1 vol.% Triton X-100 and 2 vol.% normal donkey serum in 1X PBS (the slice blocking buffer). After the primary antibody incubation, the slices were washed four times in the slice blocking buffer, for 30 min each time. The slices were then incubated for 12 h with 10 μg mL^−1^ DNA-labeled donkey anti-chicken secondary antibody in the hybridization buffer supplemented with 0.1 vol.% Triton X-100. After the secondary antibody incubation, slices were washed four times in the slice blocking buffer, for 30 min each time. The slices were then incubated for 12 h with 0.5 ng mL^−1^ of the two dual-functional labels in the hybridization buffer supplemented with 0.1 vol.% Triton X-100. The slices were then washed four times in the slice blocking buffer, for 30 min each time.

### Hydrogel synthesis, digestion, and expansion

The hydrogel solution was prepared as described in Supplementary Table 2. For stained cells, hydrogel solution was directly applied to the chambered cover glass containing the stained cells and incubated in a humidified incubator at 37 °C for 2 h. For brain slices, the stained slices were incubated at 4 °C with two changes of hydrogel solutions, for 15 min each. The slices were then placed between two cover glasses, separated by #1 cover glasses as spacers, and incubated in a humidified incubator at 37 °C for 2 h.

Proteinase K solution was prepared by diluting Proteinase K stock (New England Biolabs) 1:100 to 8 units mL^−1^ in a digestion buffer that contained 50 mM Tris (pH 8.0), 1 mM ethylenediaminetetraacetic acid (EDTA), 0.5 vol.% Triton X-100, and 0.8 M guanidine HCl. For cells, the chamber walls of the cover glasses were removed and the cover glasses with gels were incubated in the Proteinase K solution for 12 g at room temperature. For brain slices, the top cover glasses were carefully removed and the bottom cover glasses with gels were incubated in the Proteinase K solution for 12 h at room temperature. Digested cellular matter and matrices were washed in de-ionized water three times, for 2 h, 2 h, and 12 h, respectively.

### Specifications of the microscopes

The numerical aperture (NA) of the mini-microscope was calculated as approximately 0.32 at 10X magnification and 0.50 at 20X magnification. The lateral resolution of the mini-microscope was measured as approximately 1–2 μm (Supplementary Fig. 1). The point spread function (PSF) at 20X was also determined for the mini-microscope and the axial resolution was calculated as approximately 34 μm, based on the FWHM measurement (Supplementary Fig. 4). The distortion fields of the mini-microscope at 10X are shown in Supplementary Fig. 5, which were negligible in both directions. The benchtop microscope used for imaging was a Zeiss Axio Observer D1 microscope equipped with an AxioCam MRm Rev.3 camera (Carl Zeiss), an LD A-Plan 10X objective with a 0.25 NA, and an LD Plan-Neofluar 20X objective with a 0.4 NA. The confocal microscope was CSU-X1 (Yokogawa) equipped with a Zyla 5.5 camera (Andor) and a 20X objective with an NA of 1.15. A true background-free image of a stained mouse brain sample obtained from the confocal microscope in comparison with the benchtop microscope and mini-microscope is provided in Supplementary Fig. 5.

## Additional Information

**How to cite this article**: Zhang, Y. S. *et al.* Hybrid Microscopy: Enabling Inexpensive High-Performance Imaging through Combined Physical and Optical Magnifications. *Sci. Rep.* **6**, 22691; doi: 10.1038/srep22691 (2016).

## Supplementary Material

Supplementary Information
