# Supplementary material for: Hybrid Microscopy: Enabling Inexpensive High-Performance Imaging through Combined Physical and Optical Magnifications
Source: Sci Rep. 2016 Mar 15;6:22691. doi: 10.1038/srep22691 (PMC4792139; doi:10.1038/srep22691)
Supplement: Supplementary Information [file srep22691-s1.pdf]

Supplementary Information

# Hybrid Microscopy: Enabling Inexpensive High-Performance Imaging through Combined Physical and Optical Magnifications

Yu Shrike Zhang[1,2,3,†], Jae-Byum Chang[4,†], Mario Moisés Alvarez[1,2,5,6], Grissel Trujillo-de Santiago[1,2,5,6], Julio Aleman[1,2], Byambaa Batzaya[1,2], Vaishali Krishnadoss[1,2,7], Aishwarya Aravamudhan Ramanujam[1,2,7], Mehdi Kazemzadeh-Narbat[1,2], Fei Chen[8], Paul W. Tillberg[9], Mehmet Remzi Dokmeci[1,2,3], Edward S. Boyden[4,8,10,11,12,*], & Ali Khademhosseini[1,2,3,13,14,*]

[1]Biomaterials Innovation Research Center, Division of Biomedical Engineering, Department of Medicine, Brigham and Women's Hospital, Harvard Medical School, Boston 02139, MA, USA

[2]Harvard-MIT Division of Health Sciences and Technology, Cambridge 02139, MA, USA

[3]Wyss Institute for Biologically Inspired Engineering, Harvard University, Boston 02115, MA, USA

[4]Media Lab, MIT, Cambridge 02139, MA, USA

[5]Centro de Biotecnología-FEMSA, Tecnológico de Monterrey at Monterrey, CP 64849, Monterrey, Nuevo León, México

[6]Microsystems Technologies Laboratories, MIT, Cambridge, 02139, MA, USA

[7]School of Chemical & Biotechnology, SASTRA University, Tamil Nadu 613401, India

[8]Department of Biological Engineering, MIT, Cambridge 02139, MA, USA

[9]Department of Electrical Engineering and Computer Science, MIT, Cambridge 02139, MA, USA

[10]McGovern Institute, MIT, Cambridge 02139, MA, USA

[11]Department of Brain and Cognitive Sciences, MIT, Cambridge 02139, MA, USA

[12]Center for Neurobiological Engineering, MIT, Cambridge 02139, MA, USA

[13]Department of Bioindustrial Technologies, College of Animal Bioscience and Technology, Konkuk University, Hwayang-dong, Gwangjin-gu, Seoul 143-701, Republic of Korea

[14]Department of Physics, King Abdulaziz University, Jeddah 21569, Saudi Arabia

*Corresponding authors. E-mails: esb@media.mit.edu; alik@rics.bwh.harvard.edu.

[†]Y. S. Zhang and J.-B. Chang contributed equally to this work.

**Supplementary Table 1.** Hydrogel solution recipe.

| | Stock concentration | Amount added for plated cells and bacteria (μL) | Amount added for brain slices (μL) |
|---|---|---|---|
| Sodium acrylate | 33% (w/w) | 227 | 227 |
| Acrylamide | 50% (w/w) | 50 | 50 |
| N,N′-Methylenebisacrylamide | 2% (w/w) | 75 | 75 |
| Sodium chloride | 5 M | 400 | 400 |
| PBS | 10x | 100 | 100 |
| 4-hydroxy-2,2,6,6-tetramethylpiperidin-1-oxyl | 1% (w/w) | 0 | 10 |
| Ammonium persulfate | 10% (w/w) | 20 | 20 |
| Tetramethylethylenediamine | 10% (v/v) | 20 | 20 |
| Deionized water | | 108 | 98 |
| Final volume | | 1000 | 1000 |

**Supplementary Table 2.** Cost analysis of one single sample preparation process at a working volume of 100 µL, which is sufficiently large for observation using the mini-microscope.

| Product Name | Vendor | Product number | Price ($) | Amount | Unit | Amount used / single sample | Cost / single sample ($) |
|---|---|---|---|---|---|---|---|
| **Antibody and DNA** | | | | | | | |
| Primary antibody | Millipore | ab16901 | 285.00 | 250 | µL | 1 | 1.14 |
| Second antibody | Jackson ImmunoResearch Laboratory | 703-005-155 | 68.00 | 1000 | µL | 1 | 0.068 |
| DNA, for antibody conjugation | IDT | N/A | 118.00 | 665 | nmol | 0.067 | 0.012 |
| DNA, tertiary linker 1 | IDT | N/A | 616.5 | 60.4 | nmol | 0.007 | 0.069 |
| DNA, tertiary linker 2 | IDT | N/A | 616.5 | 72.6 | nmol | 0.007 | 0.057 |
| **Reagents for DNA antibody conjugation** | | | | | | | |
| Desalting column | ThermoFisher | 89882 | 122.00 | 25 | EA | 0.02* | 0.098 |
| Centrifugal concentrator | Sigma | Z614009 | 126.30 | 25 | EA | 0.01** | 0.051 |
| Centrifugal filter | Sigma | Z648043 | 133.00 | 24 | EA | 0.01** | 0.055 |
| Sulfo-S-4FB Crosslinker | Solulink | S-1008-010 | 265.00 | 10 | mg | 0.0004 | 0.009 |
| S-HyNic Crosslinker | Solulink | S-1002-105 | 165.00 | 5 | mg | 0.00006 | 0.002 |
| **Chemicals for gelation** | | | | | | | |
| Sodium acrylate | Sigma | 408220 | 58.80 | 25 | g | 0.008 | 0.018 |
| Acrylamide | Sigma | A9099 | 63.30 | 100 | g | 0.003 | 0.002 |
| N,N′-Methylenebisacrylamide | Sigma | M7279 | 49.30 | 25 | g | 0.0002 | 0.0003 |
| Ammonium Persulfate | Sigma | A3678 | 28.30 | 25 | g | 0.0002 | 0.0002 |
| N,N,N′,N′-Tetramethylethylenediamine | Sigma | T7024 | 37.90 | 25 | mL | 0.0002 | 0.0003 |
| 4-Hydroxy-TEMPO | Sigma | 176141 | 27.50 | 1 | g | 0.0001 | 0.003 |
| **Chemicals for immunostaining** | | | | | | | |
| Dextran Sulfate | Millipore | S4030 | 138.00 | 100 | mL | 0.04 | 0.055 |
| SSC | Life Technologies | 15557 | 45.00 | 1000 | mL | 0.02 | 0.001 |
| Yeast tRNA | Roche | 10109495001 | 127.00 | 100 | mg | 0.2 | 0.254 |
| Normal Donkey | Jackson | 017-000- | 20.00 | 2 | mL | 0.04 | 0.4 |

| | | | | | | | |
|---|---|---|---|---|---|---|---|
| Serum | Immunoresearch | 001 | | | | | |
| **Chemicals for digestion** | | | | | | | |
| Proteinase K | New England Biolabs | P8107S | 73.00 | 2 | mL | 0.001 | 0.037 |
| Ethylenediaminetetraacetic acid | Sigma | EDS | 21.40 | 100 | g | 0.00003 | 0.000006 |
| Guanidine HCl | Sigma | G3272 | 34.90 | 25 | g | 0.0077 | 0.011 |
| Tris-HCl, 1M pH 8.0 | Life Technologies | AM9855 | 49.00 | 100 | mL | 0.005 | 0.0002 |
| **Chemicals for fixation** | | | | | | | |
| Paraformaldehyde | Electron Microscopy Sciences | 15710 | 26.00 | 100 | mL | 0.025 | 0.007 |
| Triton X-100 | Sigma | X100 | 35.60 | 100 | mL | 0.0004 | 0.0001 |
| Glycine | Sigma | 50046 | 19.20 | 50 | g | 0.0023 | 0.0009 |
| PBS (10x) | Life Technologies | 70011-044 | 40.00 | 500 | mL | 0.17 | 0.014 |
| **Total** | | | | | | | **2.365** |

* Two columns are used to conjugate 100 µL secondary antibody with DNA.

** One concentrator and filter are used to conjugate 100 µL secondary antibody with DNA.

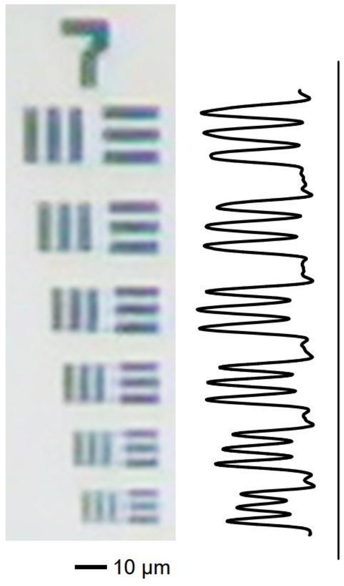


**Supplementary Figure 1: Resolution determination of mini-microscope**. Mini-microscope image of Group 7 resolution target and line profiles showing clear separation of the peaks between adjacent lines. The thinnest line width at the bottom is 2.19 µm.

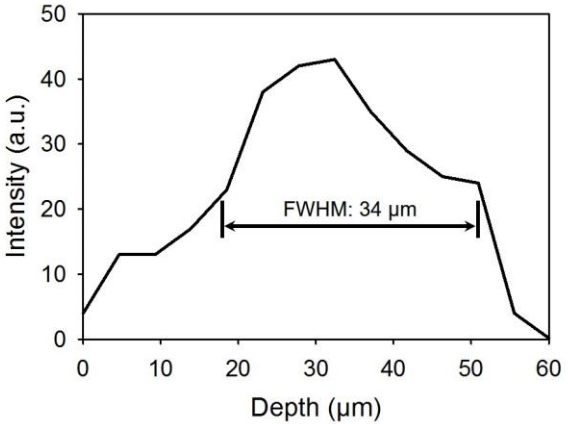


**Supplementary Figure 2: Measurement of the PSF of the mini-microscope.** The axial resolution of the mini-microscope was characterized to be approximately 34 µm based on the FWHM.

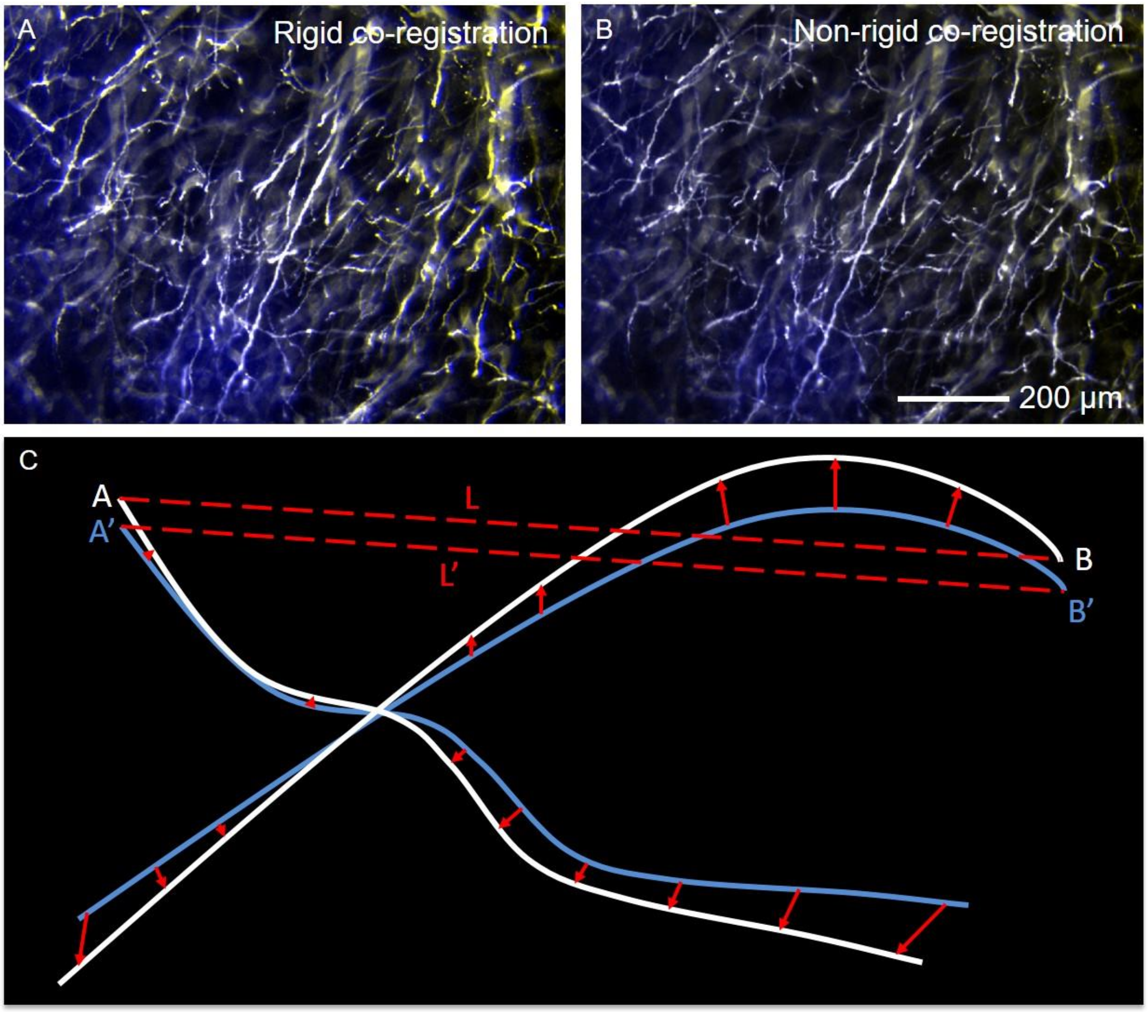


**Supplementary Figure 3: Principle of RMS error quantification.** (**A**,**B**) Rigid and non-rigid co-registration of ExMM and ExM at 10X magnification (see Fig. 4A,B). (**C**) The blue lines, representing structures in the ExMM image, are mapped to the white lines, representing the structures in the ExM image, via the vector field depicted by black arrows. Measurement L’ along the line segment A’B’ in the ExM image is mapped to measurement L along the line segment AB in the ExMM image. The ExM error is calculated as |L-L’|; i.e. the difference between the deformation vectors AA’ and BB’. The generated deformation field generated can then be used to calculate the RMS error between all extracted features in the ExMM and ExM images.

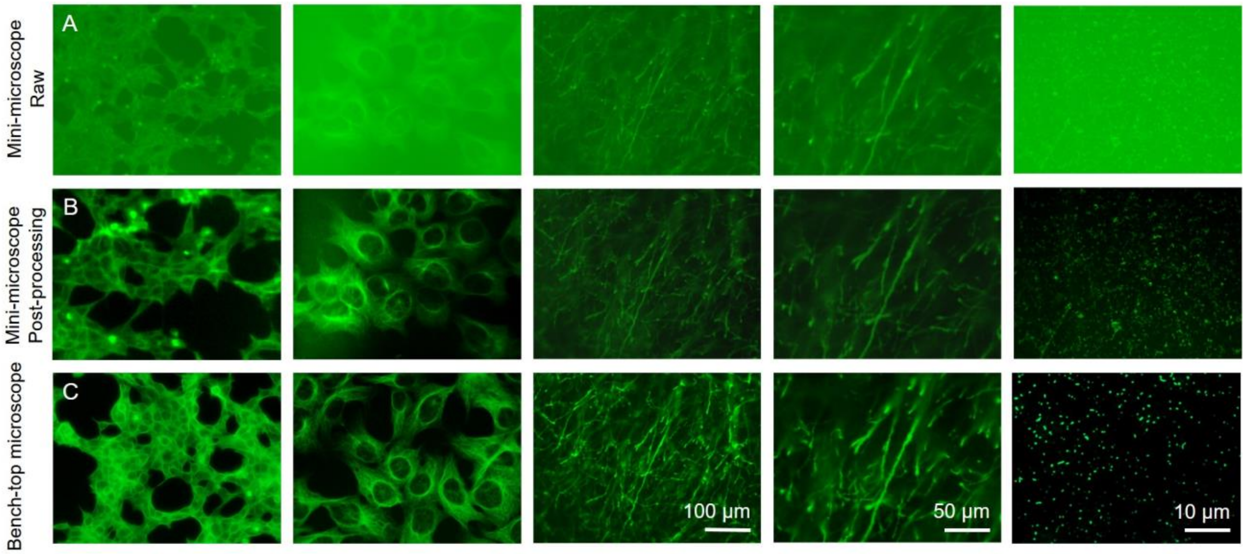


**Supplementary Figure 4: Comparison between mini-microscope and benchtop microscope images.** (**A**,**B**) Raw and processed images obtained with the mini-microscope, respectively, and (**C**) raw images obtained with the benchtop microscope.

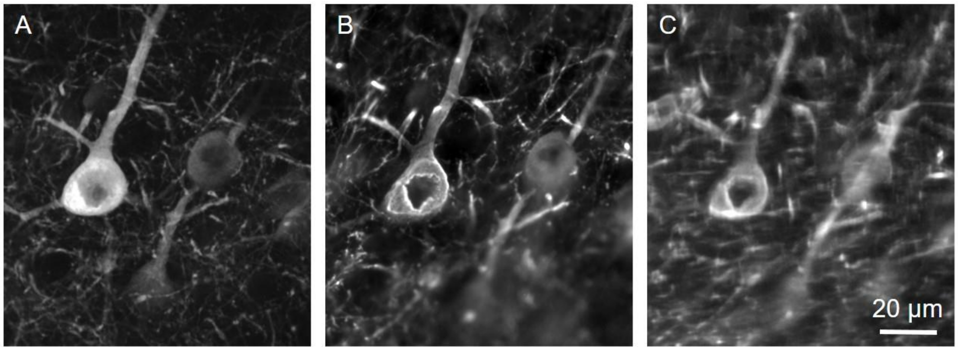


**Supplementary Figure 5: Comparison among confocal, benchtop, and mini-microscope images.** (**A**) Confocal image pre-expansion; NA=1.15 (water-immersion objective, 40X). (**B**) Benchtop microscope image post-expansion; objective: 10X, NA=0.25. (**C**) ExMM image at 10X (NA~0.32).
